# Crystal structure of 1-(3-chloro­phen­yl)piperazin-1-ium picrate–picric acid (2/1)

**DOI:** 10.1107/S1600536814023654

**Published:** 2014-10-31

**Authors:** Channappa N. Kavitha, Jerry P. Jasinski, Manpreet Kaur, Brian J. Anderson, H. S. Yathirajan

**Affiliations:** a Department of Studies in Chemistry, University of Mysore, Manasagangotri, Mysore 570 006, India; b Department of Chemistry, Keene State College, 229 Main Street, Keene, NH 03435-2001, USA

**Keywords:** crystal structure, piperazin-1-ium, picrate, picric acid, salt

## Abstract

The title salt {systematic name: bis­[1-(3-chloro­phen­yl)piperazinium 2,4,6-tri­nitro­phenolate]–picric acid (2/1)}, 2C_10_H_14_ClN_2_^+^·2C_6_H_5_N_3_O_7_^−^·C_6_H_6_N_3_O_7_, crystallized with two independent 1-(3-chloro­phen­yl)piperazinium cations, two picrate anions and a picric acid mol­ecule in the asymmetric unit. The six-membered piperazine ring in each cation adopts a slightly distorted chair conformation and contains a protonated N atom. In the picric acid mol­ecule, the mean planes of the nitro groups in the *ortho*-, *meta*-, and *para*-positions are twisted from the benzene ring by 31.5 (3), 7.7 (1), and 3.8 (2)°, respectively. In the anions, the dihedral angles between the benzene ring and the *ortho*-, *meta*-, and *para*-nitro groups are 36.7 (1), 5.0 (6), 4.8 (2)°, and 34.4 (9), 15.3 (8), 4.5 (1)°, respectively. The nitro group in one anion is disordered and was modeled with two sites for one O atom with an occupancy ratio of 0.627 (7):0.373 (7). In the crystal, the picric acid mol­ecule inter­acts with the picrate anion through a trifurcated O—H⋯O four-centre hydrogen bond involving an intra­molecular O—H⋯O hydrogen bond and a weak C—H⋯O inter­action. Weak inter­molecular C—H⋯O inter­actions are responsible for the formation of cation–anion–cation trimers resulting in a chain along [010]. In addition, weak C—H⋯Cl and weak π–π inter­actions [centroid–centroid distances of 3.532 (3), 3.756 (4) and 3.705 (3) Å] are observed and contribute to the stability of the crystal packing.

## Related literature   

For related structures, see: Homrighausen *et al.* (2002 ▶); Koysal *et al.* (2003 ▶). For the biological activity of piperazine derivatives, see: Berkheij *et al.* (2005 ▶); Humle & Cherrier (1999 ▶); Kennett & Curzon (1988 ▶); Petkov *et al.* (1995 ▶).
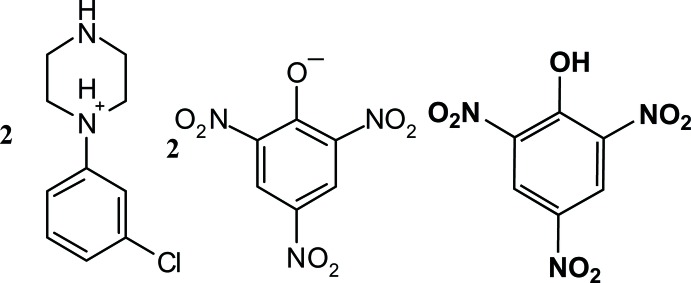


## Experimental   

### Crystal data  

2C_10_H_14_ClN_2_^+^·2C_6_H_2_N_3_O_7_^−^·C_6_H_3_N_3_O_7_
*M* *_r_* = 1080.69
Monoclinic, 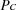

*a* = 11.2213 (6) Å
*b* = 14.6239 (7) Å
*c* = 14.1804 (8) Å
β = 104.405 (5)°
*V* = 2253.8 (2) Å^3^
*Z* = 2
Mo *K*α radiation
μ = 0.24 mm^−1^
*T* = 173 K
0.48 × 0.46 × 0.38 mm

### Data collection  

Agilent Eos Gemini diffractometer
Absorption correction: multi-scan (*CrysAlis PRO* and *CrysAlis RED*; Agilent, 2012 ▶) *T*_min_ = 0.598, *T*_max_ = 1.000
28568 measured reflections
13782 independent reflections
10981 reflections with *I* > 2σ(*I*)
*R*_int_ = 0.035

### Refinement  

*R*[*F* ^2^ > 2σ(*F* ^2^)] = 0.054
*wR*(*F* ^2^) = 0.139
*S* = 1.02
13782 reflections
671 parameters
14 restraints
H-atom parameters constrained
Δρ_max_ = 0.65 e Å^−3^
Δρ_min_ = −0.49 e Å^−3^
Absolute structure: Flack *x* determined using 4095 quotients [(*I* ^+^)−(*I* ^−^)]/[(*I* ^+^)+(*I* ^−^)] (Parsons *et al.*, 2013 ▶)
Absolute structure parameter: 0.09 (3)

### 

Data collection: *CrysAlis PRO* (Agilent, 2012 ▶); cell refinement: *CrysAlis PRO*; data reduction: *CrysAlis RED* (Agilent, 2012 ▶); program(s) used to solve structure: *SUPERFLIP* (Palatinus & Chapuis, 2007 ▶); program(s) used to refine structure: *SHELXL2012* (Sheldrick, 2008 ▶); molecular graphics: *OLEX2* (Dolomanov *et al.*, 2009 ▶); software used to prepare material for publication: *OLEX2*.

## Supplementary Material

Crystal structure: contains datablock(s) I. DOI: 10.1107/S1600536814023654/bv2237sup1.cif

Structure factors: contains datablock(s) I. DOI: 10.1107/S1600536814023654/bv2237Isup2.hkl

Supporting information file. DOI: 10.1107/S1600536814023654/bv2237Isup3.cml

**Fig. 1.**
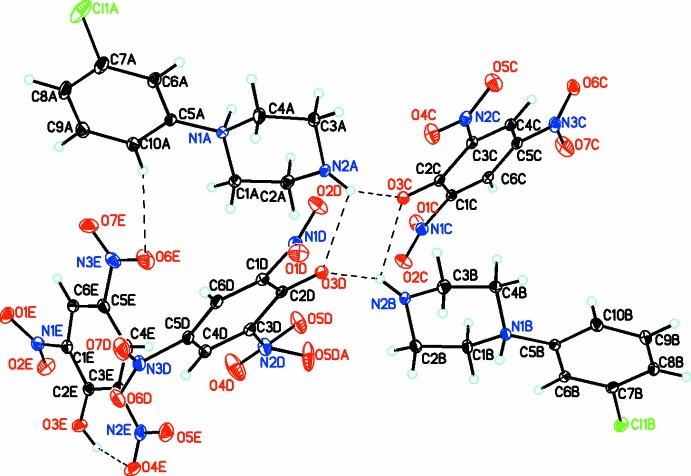
ORTEP drawing of (I) (2 C10H14ClN2+ .2 C6H5N3O7- . C6H6N3O7) showing the labeling scheme of the molecule with 30% probability displacement ellipsoids. Dashed lines indicate intramolecular O—H···O and bifurcated N—H···O hydrogen bonds and a weak C—H···O intermolecular interaction.

**Fig. 2.**
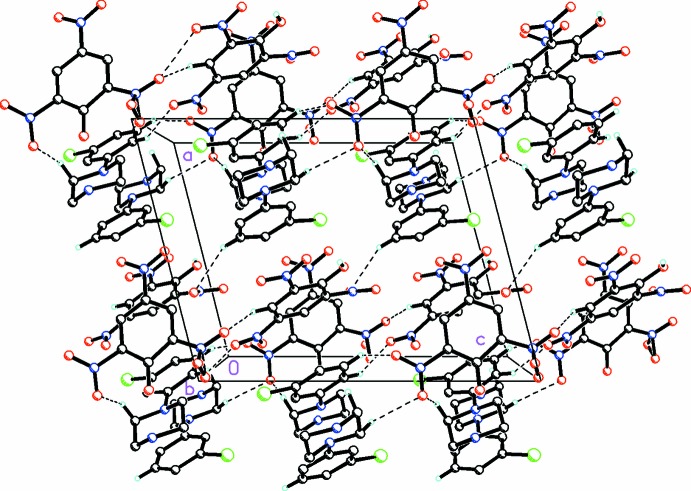
Molecular packing for (I) viewed along the b axis. Dashed lines indicate a trifurcated O—H···O four centre hydrogen bond involving a O3E—H3E···O4E intramolecular hydrogen bond and a weak C—H···O interaction with that of picrate anion. Weak C—H···O interactions are responsible for the formation of cation-anion-cation trimers resulting in a 1D chain along [0 1 0]. H atoms not involved in hydrogen bonding have been removed for clarity.

10 14 2 + 6 5 3 7 − 6 6 3 7 . DOI: 10.1107/S1600536814023654/bv2237fig1.tif
ORTEP drawing of (I) (2 C_10_H_14_ClN_2_^+^. 2 C_6_H_5_N_3_O_7_^−^. C_6_H_6_N_3_O_7_) showing the labeling scheme of the mol­ecule with 30% probability displacement ellipsoids. Dashed lines indicate intra­molecular O—H⋯O and bifurcated N—H⋯O hydrogen bonds and a weak C—H⋯O inter­molecular inter­action.

b E E E . DOI: 10.1107/S1600536814023654/bv2237fig2.tif
Mol­ecular packing for (I) viewed along the *b* axis. Dashed lines indicate a trifurcated O—H⋯O four centre hydrogen bond involving a O3*E*—H3*E*⋯O4*E* intra­molecular hydrogen bond and a weak C—H⋯O inter­action with that of picrate anion. Weak C—H⋯O inter­actions are responsible for the formation of cation-anion-cation trimers resulting in a 1D chain along [0 1 0]. H atoms not involved in hydrogen bonding have been removed for clarity.

CCDC reference: 1031336

Additional supporting information:  crystallographic information; 3D view; checkCIF report

## Figures and Tables

**Table 1:** Hydrogen-bond geometry (, )

*D*H*A*	*D*H	H*A*	*D* *A*	*D*H*A*
N1*A*H1*A*Cl1*B* ^i^	1.00	2.78	3.665(3)	147
N2*A*H2*A*O3*C*	0.88	2.29	2.723(4)	110
N2*A*H2*A*O3*D*	0.88	2.34	2.787(4)	112
C3*A*H3*AB*O7*D* ^ii^	0.99	2.63	3.451(5)	140
C4*A*H4*AA*Cl1*B* ^iii^	0.99	2.88	3.690(4)	139
C4*A*H4*AB*O2*D*	0.99	2.51	3.253(5)	132
C8*A*H8*A*O7*C* ^iv^	0.95	2.64	3.572(5)	168
C10*A*H10*A*O6*E* ^v^	0.95	2.53	3.400(5)	152
N1*B*H1*B*Cl1*A* ^vi^	1.00	2.80	3.653(3)	143
N2*B*H2*B*O3*C*	0.88	2.38	2.848(4)	114
N2*B*H2*B*O3*D*	0.88	2.34	2.771(4)	111
C1*B*H1*BA*O2*C*	0.99	2.66	3.375(5)	129
C1*B*H1*BB*O2*D* ^vii^	0.99	2.52	3.317(4)	137
C2*B*H2*BA*O7*C* ^viii^	0.99	2.56	3.419(5)	145
C4*B*H4*BA*O1*C* ^iii^	0.99	2.63	3.232(4)	120
C6*C*H6*C*O5*C* ^vii^	0.95	2.48	3.310(4)	146
O3*E*H3*E*O4*C*	0.84	2.51	3.037(4)	122
O3*E*H3*E*O5*C*	0.84	2.43	2.962(4)	122
O3*E*H3*E*O4*E*	0.84	1.86	2.565(4)	140
O3*E*H3*E*N2*E*	0.84	2.47	2.899(4)	113
C4*E*H4*E*O4*D* ^ix^	0.95	2.57	3.412(6)	148

Symmetry codes: (i) 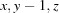; (ii) 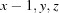; (iii) 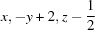; (iv) 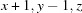; (v) 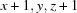; (vi) 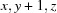; (vii) 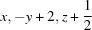; (viii) 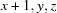; (ix) 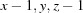.

## supplementary crystallographic information

### S1. Comment

Piperazine derived designer drug 1-(3-chlorophenyl)piperazine or meta-chlorophenylpiperazine (mCPP), a psychoactive drug of the phenylpiperazine class, is known to induce headaches in humans and has been used for testing potential antimigraine medications (Petkov *et al.*, 1995). In addition, it has potent anorectic effects and has encouraged the development of selective 5-HT2C receptor agonists for the treatment of obesity (Kennett & Curzon, 1988). It is a major metabolite of the psychotropic drugs trazodone and nefazodone. Also piperazine derivatives are found in biologically active compounds across a number of different therapeutic areas (Berkheij *et al.*, 2005) such as antifungal, antibacterial, antimalarial, antipsychotic, antidepressant and antitumour activity against colon, prostate, breast, lung and leukemia tumors (Humle & Cherrier, 1999). The crystal structures of some related compounds viz., 1-(3-chlorophenyl)-4-(3-chloropropyl)piperazinium chloride (Homrighausen *et al.*, 2002), 3-[4-(2-chlorophenyl)piperazinomethyl]-5-methyl-1-benzoxazolin-2(3H)-one (Koysal *et al.*, 2003), have been reported. In view of the importance of piperazines, this paper reports the crystal and molecular structure of the title compound, (I), 2 C_10_H_14_ClN_2_^+^ . 2 C_6_H_5_N_3_O_7_^-^ . C_6_H_6_N_3_O_7_.

The title compound, (I), crystallizes with two independent 1-(3-chlorophenyl)piperazinium cations (A and B), two picrate anions (C and D) and a picric acid molecule (E) in the asymmetric unit (Fig. 1).The six-membered piperazine ring in each cation adopts a slightly distorted chair conformation with puckering parameters Q, θ, and φ = 0.5422 Å, 5.8 (2)° and 148.01 (21)° (A); Q, θ, and φ = 0.5515 Å, 176.0 (7)° and 38.66 (04)° (B) and contains a protonated N atom . In the picric acid molecule (E), the mean planes of the nitro groups at positions 2, 4 and 6 are twisted from the mean plane of the phenyl ring by 31.5 (3)°, 7.7 (1)° and 3.8 (2)°, respectively. The dihedral angles between the mean planes of the phenyl ring and nitro groups at positions 2,4 and 6 are 36.7 (1)°, 5.0 (6)°, 4.8 (2)° and 34.4 (9)°, 15.3 (8)°, 4.5 (1)° in anions D and E, respectively. Disorder was modeled over two sets of sites for the O5D oxygen of the nitro group in anion D with an occupancy ratio of 0.53 (3) : 0.47 (3). Bond lengths are in normal ranges. In the crystal, the picric acid molecule (E) interacts with the picrate anion (C) through a trifurcated O—H···O four centre hydrogen bond involving an O3E—H3E···O4E intramolecular hydrogen bond and a weak C—H···O intermolecular interaction with that of picrate anion (D) (Fig. 2). Additional weak C—H···O intermolecular interactions are responsible for the formation of cation-anion-cation trimers resulting in a 1D chain along [0 1 0] (Fig. 2). In addition, weak Cg1–Cg2, Cg3—Cg5 and Cg5—Cg7 π—π intermolecular interactions are observed and contribute to crystal packing stability (Cg1–Cg2 = 3.532 (3) Å, x, y, z ; Cg3—Cg5 = 3.756 (4) Å, -1+x, y, z ; 1+x, y, z ; Cg5—Cg7 = 3.705 (3) Å, x, 1-y, 1/z +z; Cg1 = C1D–C6D; Cg2 = C1C–C6C; Cg3 = C1E–C6E; Cg5 = C5A–C10A; Cg7 = C5B–C10B).

### S2. Experimental

1-(3-Chlorophenyl)piperazine hydrochloride (2.31 g, 0.01 mol) and picric acid (2.29 g, 0.01 mol) were dissolved in hot methanol and stirred over a heating magnetic stirrer for few minutes . The resulting solution was allowed to cool slowly at room temperature. X-ray quality crystals of the title compound appeared in a day. (M.P.: 408–413 K).

### S3. Refinement

All of the H atoms were placed in their calculated positions and then refined using the riding model with Atom—H lengths of 0.95Å (CH); 0.99Å (CH_2_); 0.88Å, 1.00Å (NH) or 0.84Å (OH). Isotropic displacement parameters for these atoms were set to 1.2 (CH, CH_2_, NH) or 1.5 (OH) times *U*_eq_ of the parent atom. Idealized tetrahedral OH was refined as a rotating group, O3E(H3E). Disorder was modeled over two sets of sites for the O5D oxygen of the nitro group in anion D with an occupancy ratio of 0.627 (7):0.373 (7)

### Crystal data

**Table:**

2C_10_H_14_ClN_2_^+^·2C_6_H_2_N_3_O_7_^−^·C_6_H_3_N_3_O_7_	*F*(000) = 1112
*M**_r_* = 1080.69	*D*_x_ = 1.592 Mg m^−^^3^
Monoclinic, *P**c*	Mo *K*α radiation, λ = 0.71073 Å
*a* = 11.2213 (6) Å	Cell parameters from 7430 reflections
*b* = 14.6239 (7) Å	θ = 3.3–32.8°
*c* = 14.1804 (8) Å	µ = 0.24 mm^−^^1^
β = 104.405 (5)°	*T* = 173 K
*V* = 2253.8 (2) Å^3^	Irregular, violet
*Z* = 2	0.48 × 0.46 × 0.38 mm

### Data collection

**Table:**

Agilent Eos Gemini diffractometer	10981 reflections with *I* > 2σ(*I*)
Detector resolution: 16.0416 pixels mm^-1^	*R*_int_ = 0.035
ω scans	θ_max_ = 32.9°, θ_min_ = 3.3°
Absorption correction: multi-scan (*CrysAlis PRO* and *CrysAlis RED*; Agilent, 2012)	*h* = −16→16
*T*_min_ = 0.598, *T*_max_ = 1.000	*k* = −21→18
28568 measured reflections	*l* = −17→21
13782 independent reflections	

### Refinement

**Table:**

Refinement on *F*^2^	Hydrogen site location: mixed
Least-squares matrix: full	H-atom parameters constrained
*R*[*F*^2^ > 2σ(*F*^2^)] = 0.054	*w* = 1/[σ^2^(*F*_o_^2^) + (0.0686*P*)^2^ + 0.4633*P*] where *P* = (*F*_o_^2^ + 2*F*_c_^2^)/3
*wR*(*F*^2^) = 0.139	(Δ/σ)_max_ = 0.001
*S* = 1.02	Δρ_max_ = 0.65 e Å^−^^3^
13782 reflections	Δρ_min_ = −0.49 e Å^−^^3^
671 parameters	Absolute structure: Flack *x* determined using 4095 quotients [(*I*^+^)-(*I*^-^)]/[(*I*^+^)+(*I*^-^)] (Parsons *et al.*, 2013)
14 restraints	Absolute structure parameter: 0.09 (3)
Primary atom site location: structure-invariant direct methods	

### Special details

**Table:**

Geometry. All esds (except the esd in the dihedral angle between two l.s. planes) are estimated using the full covariance matrix. The cell esds are taken into account individually in the estimation of esds in distances, angles and torsion angles; correlations between esds in cell parameters are only used when they are defined by crystal symmetry. An approximate (isotropic) treatment of cell esds is used for estimating esds involving l.s. planes.

### Fractional atomic coordinates and isotropic or equivalent isotropic displacement parameters (Å^2^)

**Table:**

	*x*	*y*	*z*	*U*_iso_*/*U*_eq_	Occ. (<1)
Cl1A	0.92045 (14)	0.25015 (8)	0.65989 (11)	0.0641 (4)	
N1A	0.8303 (2)	0.55334 (18)	0.8092 (2)	0.0248 (5)	
H1A	0.7508	0.5351	0.8237	0.030*	
N2A	0.7416 (3)	0.73801 (19)	0.7774 (2)	0.0268 (5)	
H2A	0.7475	0.7963	0.7638	0.032*	
C1A	0.8725 (3)	0.6246 (2)	0.8820 (2)	0.0299 (7)	
H1AA	0.9487	0.6525	0.8717	0.036*	
H1AB	0.8924	0.5967	0.9477	0.036*	
C2A	0.7768 (3)	0.6980 (2)	0.8767 (2)	0.0307 (7)	
H2AA	0.7032	0.6715	0.8931	0.037*	
H2AB	0.8099	0.7465	0.9248	0.037*	
C3A	0.6952 (3)	0.6651 (2)	0.7044 (3)	0.0337 (7)	
H3AA	0.6766	0.6919	0.6383	0.040*	
H3AB	0.6178	0.6398	0.7152	0.040*	
C4A	0.7874 (4)	0.5890 (3)	0.7109 (2)	0.0356 (8)	
H4AA	0.7493	0.5387	0.6668	0.043*	
H4AB	0.8587	0.6119	0.6885	0.043*	
C5A	0.8936 (3)	0.4696 (2)	0.8210 (2)	0.0260 (6)	
C6A	0.8836 (3)	0.4097 (2)	0.7423 (3)	0.0306 (7)	
H6A	0.8392	0.4275	0.6789	0.037*	
C7A	0.9392 (4)	0.3242 (2)	0.7578 (3)	0.0391 (9)	
C8A	1.0081 (4)	0.2962 (3)	0.8474 (4)	0.0457 (10)	
H8A	1.0459	0.2376	0.8561	0.055*	
C9A	1.0203 (4)	0.3564 (3)	0.9246 (4)	0.0449 (10)	
H9A	1.0677	0.3389	0.9872	0.054*	
C10A	0.9648 (3)	0.4418 (3)	0.9123 (3)	0.0350 (8)	
H10A	0.9752	0.4819	0.9664	0.042*	
Cl1B	0.62724 (9)	1.49001 (6)	0.96487 (7)	0.0378 (2)	
N1B	0.7345 (2)	1.18462 (18)	0.82700 (19)	0.0239 (5)	
H1B	0.8144	1.1999	0.8115	0.029*	
N2B	0.8215 (3)	1.00073 (19)	0.8686 (2)	0.0271 (5)	
H2B	0.8156	0.9433	0.8853	0.033*	
C1B	0.7757 (3)	1.1532 (2)	0.9275 (2)	0.0292 (7)	
H1BA	0.7039	1.1317	0.9501	0.035*	
H1BB	0.8133	1.2049	0.9694	0.035*	
C2B	0.8692 (3)	1.0758 (2)	0.9376 (2)	0.0306 (7)	
H2BA	0.9463	1.0998	0.9251	0.037*	
H2BB	0.8883	1.0519	1.0050	0.037*	
C3B	0.7848 (3)	1.0360 (2)	0.7668 (2)	0.0302 (7)	
H3BA	0.7507	0.9854	0.7217	0.036*	
H3BB	0.8579	1.0606	0.7482	0.036*	
C4B	0.6894 (3)	1.1104 (2)	0.7587 (2)	0.0286 (6)	
H4BA	0.6679	1.1349	0.6915	0.034*	
H4BB	0.6139	1.0843	0.7721	0.034*	
C5B	0.6729 (3)	1.2684 (2)	0.8106 (2)	0.0220 (5)	
C6B	0.6776 (3)	1.3311 (2)	0.8870 (2)	0.0239 (6)	
H6B	0.7206	1.3157	0.9516	0.029*	
C7B	0.6197 (3)	1.4149 (2)	0.8677 (2)	0.0265 (6)	
C8B	0.5555 (3)	1.4419 (2)	0.7758 (3)	0.0304 (7)	
H8B	0.5157	1.4997	0.7648	0.036*	
C9B	0.5519 (3)	1.3805 (3)	0.7003 (3)	0.0325 (7)	
H9B	0.5100	1.3971	0.6358	0.039*	
C10B	0.6080 (3)	1.2957 (2)	0.7169 (2)	0.0289 (6)	
H10B	0.6025	1.2550	0.6638	0.035*	
O1C	0.4915 (3)	0.9428 (2)	1.05874 (19)	0.0454 (7)	
O2C	0.6523 (3)	0.9650 (2)	1.00322 (19)	0.0452 (7)	
O3C	0.6151 (2)	0.88075 (17)	0.82868 (18)	0.0296 (5)	
O4C	0.5437 (3)	0.8427 (2)	0.6392 (2)	0.0467 (7)	
O5C	0.3560 (3)	0.8683 (2)	0.56305 (19)	0.0466 (7)	
O6C	0.0627 (2)	0.9996 (2)	0.7057 (2)	0.0401 (6)	
O7C	0.1213 (3)	1.0683 (3)	0.8433 (3)	0.0700 (12)	
N1C	0.5411 (3)	0.9534 (2)	0.9911 (2)	0.0299 (6)	
N2C	0.4405 (3)	0.8721 (2)	0.6368 (2)	0.0295 (6)	
N3C	0.1412 (3)	1.0204 (2)	0.7786 (2)	0.0347 (7)	
C1C	0.4624 (3)	0.9527 (2)	0.8926 (2)	0.0223 (5)	
C2C	0.5083 (3)	0.9130 (2)	0.8151 (2)	0.0229 (6)	
C3C	0.4156 (3)	0.9126 (2)	0.7237 (2)	0.0221 (5)	
C4C	0.2976 (3)	0.9478 (2)	0.7118 (2)	0.0243 (6)	
H4C	0.2404	0.9464	0.6499	0.029*	
C5C	0.2653 (3)	0.9847 (2)	0.7909 (2)	0.0246 (6)	
C6C	0.3456 (3)	0.9867 (2)	0.8819 (2)	0.0244 (6)	
H6C	0.3207	1.0110	0.9361	0.029*	
O1D	1.0791 (3)	0.7969 (2)	0.59714 (19)	0.0411 (6)	
O2D	0.9161 (3)	0.7703 (2)	0.6472 (2)	0.0480 (7)	
O3D	0.9480 (2)	0.85104 (16)	0.8231 (2)	0.0324 (5)	
O4D	1.1744 (4)	0.8446 (4)	1.0946 (3)	0.0869 (15)	
O5D	1.0035 (6)	0.8857 (5)	1.0074 (4)	0.0618 (17)	0.627 (7)
O5DA	1.0678 (10)	0.9384 (9)	1.0013 (6)	0.0618 (17)	0.373 (7)
O6D	1.4946 (2)	0.7214 (2)	0.9614 (2)	0.0466 (7)	
O7D	1.4511 (3)	0.6764 (2)	0.8116 (2)	0.0536 (8)	
N1D	1.0278 (3)	0.7829 (2)	0.6619 (2)	0.0304 (6)	
N2D	1.1137 (3)	0.8616 (2)	1.0141 (2)	0.0380 (7)	
N3D	1.4227 (3)	0.7115 (2)	0.8814 (2)	0.0342 (7)	
C1D	1.1031 (3)	0.7794 (2)	0.7616 (2)	0.0239 (6)	
C2D	1.0545 (3)	0.81867 (19)	0.8375 (2)	0.0217 (5)	
C3D	1.1443 (3)	0.8189 (2)	0.9302 (2)	0.0238 (6)	
C4D	1.2613 (3)	0.7834 (2)	0.9455 (2)	0.0262 (6)	
H4D	1.3160	0.7845	1.0085	0.031*	
C5D	1.2975 (3)	0.7461 (2)	0.8674 (2)	0.0263 (6)	
C6D	1.2204 (3)	0.7449 (2)	0.7751 (2)	0.0256 (6)	
H6D	1.2475	0.7207	0.7219	0.031*	
O1E	0.3217 (3)	0.4304 (2)	0.5021 (2)	0.0527 (8)	
O2E	0.3337 (3)	0.5529 (2)	0.5875 (2)	0.0558 (8)	
O3E	0.4307 (3)	0.68664 (19)	0.50528 (19)	0.0400 (6)	
H3E	0.4497	0.7412	0.4978	0.060*	
O4E	0.4634 (3)	0.82228 (19)	0.3998 (2)	0.0444 (7)	
O5E	0.3773 (3)	0.8250 (2)	0.2456 (2)	0.0542 (8)	
O6E	0.1041 (3)	0.5869 (3)	0.0935 (2)	0.0641 (10)	
O7E	0.0989 (3)	0.4590 (3)	0.1698 (2)	0.0573 (9)	
N1E	0.3236 (3)	0.5143 (2)	0.5112 (2)	0.0369 (7)	
N2E	0.3960 (3)	0.7887 (2)	0.3253 (3)	0.0381 (7)	
N3E	0.1328 (3)	0.5378 (3)	0.1652 (2)	0.0450 (8)	
C1E	0.3074 (3)	0.5679 (2)	0.4215 (2)	0.0287 (6)	
C2E	0.3609 (3)	0.6551 (2)	0.4227 (2)	0.0286 (6)	
C3E	0.3377 (3)	0.7005 (2)	0.3325 (3)	0.0290 (6)	
C4E	0.2639 (3)	0.6635 (3)	0.2476 (2)	0.0325 (7)	
H4E	0.2480	0.6961	0.1878	0.039*	
C5E	0.2145 (3)	0.5779 (3)	0.2530 (2)	0.0320 (7)	
C6E	0.2367 (3)	0.5283 (2)	0.3380 (3)	0.0313 (7)	
H6E	0.2043	0.4684	0.3392	0.038*	

### Atomic displacement parameters (Å^2^)

**Table:**

	*U*^11^	*U*^22^	*U*^33^	*U*^12^	*U*^13^	*U*^23^
Cl1A	0.0931 (10)	0.0309 (5)	0.0868 (9)	0.0029 (5)	0.0572 (8)	−0.0118 (5)
N1A	0.0253 (12)	0.0190 (12)	0.0291 (13)	−0.0001 (10)	0.0049 (10)	−0.0015 (10)
N2A	0.0255 (13)	0.0186 (12)	0.0358 (14)	0.0034 (10)	0.0066 (11)	−0.0028 (10)
C1A	0.0320 (17)	0.0223 (15)	0.0309 (16)	0.0019 (13)	−0.0010 (13)	−0.0015 (12)
C2A	0.0384 (19)	0.0254 (16)	0.0289 (16)	0.0024 (13)	0.0095 (14)	−0.0039 (12)
C3A	0.0350 (18)	0.0256 (16)	0.0347 (17)	0.0082 (13)	−0.0023 (14)	−0.0030 (13)
C4A	0.046 (2)	0.0296 (17)	0.0271 (16)	0.0135 (15)	0.0007 (14)	−0.0012 (13)
C5A	0.0193 (14)	0.0195 (14)	0.0393 (17)	−0.0021 (11)	0.0071 (12)	0.0025 (12)
C6A	0.0295 (16)	0.0237 (15)	0.0428 (18)	0.0013 (12)	0.0169 (14)	0.0020 (13)
C7A	0.0363 (19)	0.0222 (16)	0.067 (3)	0.0011 (14)	0.0289 (18)	−0.0009 (16)
C8A	0.0346 (19)	0.0214 (17)	0.086 (3)	0.0046 (14)	0.024 (2)	0.0143 (18)
C9A	0.0315 (18)	0.0300 (19)	0.068 (3)	−0.0023 (15)	0.0029 (17)	0.0184 (18)
C10A	0.0314 (17)	0.0273 (17)	0.0421 (19)	−0.0006 (13)	0.0014 (14)	0.0082 (14)
Cl1B	0.0468 (5)	0.0253 (4)	0.0420 (5)	0.0016 (4)	0.0122 (4)	−0.0059 (3)
N1B	0.0239 (12)	0.0224 (12)	0.0234 (12)	0.0041 (10)	0.0020 (9)	−0.0012 (10)
N2B	0.0244 (12)	0.0204 (12)	0.0351 (14)	0.0046 (10)	0.0046 (10)	−0.0008 (10)
C1B	0.0332 (17)	0.0261 (15)	0.0246 (14)	0.0102 (13)	0.0001 (12)	−0.0018 (12)
C2B	0.0282 (16)	0.0281 (16)	0.0317 (16)	0.0055 (13)	0.0003 (12)	−0.0020 (13)
C3B	0.0345 (17)	0.0278 (16)	0.0286 (16)	0.0053 (13)	0.0088 (13)	−0.0051 (13)
C4B	0.0294 (16)	0.0243 (15)	0.0278 (15)	−0.0003 (12)	−0.0013 (12)	−0.0037 (12)
C5B	0.0189 (13)	0.0209 (13)	0.0255 (14)	−0.0006 (10)	0.0039 (10)	0.0004 (11)
C6B	0.0244 (14)	0.0203 (14)	0.0259 (14)	−0.0021 (11)	0.0040 (11)	0.0013 (11)
C7B	0.0266 (15)	0.0208 (14)	0.0329 (16)	−0.0028 (12)	0.0087 (12)	−0.0027 (12)
C8B	0.0255 (15)	0.0245 (15)	0.0388 (18)	0.0017 (12)	0.0036 (13)	0.0079 (13)
C9B	0.0308 (17)	0.0306 (17)	0.0319 (16)	0.0031 (13)	0.0001 (13)	0.0065 (13)
C10B	0.0294 (16)	0.0302 (16)	0.0240 (14)	0.0022 (12)	0.0007 (12)	0.0013 (12)
O1C	0.0474 (16)	0.063 (2)	0.0284 (12)	0.0104 (14)	0.0137 (11)	0.0045 (12)
O2C	0.0338 (14)	0.064 (2)	0.0338 (14)	−0.0053 (14)	0.0014 (11)	−0.0042 (13)
O3C	0.0242 (11)	0.0269 (12)	0.0375 (12)	0.0049 (9)	0.0076 (9)	−0.0051 (9)
O4C	0.0469 (16)	0.0616 (19)	0.0347 (14)	0.0250 (14)	0.0158 (12)	−0.0032 (13)
O5C	0.0498 (17)	0.062 (2)	0.0266 (12)	0.0103 (14)	0.0068 (11)	−0.0078 (12)
O6C	0.0238 (12)	0.0448 (16)	0.0506 (15)	0.0054 (10)	0.0071 (11)	0.0124 (12)
O7C	0.0481 (19)	0.093 (3)	0.067 (2)	0.0362 (19)	0.0112 (16)	−0.031 (2)
N1C	0.0301 (14)	0.0319 (15)	0.0272 (13)	0.0004 (11)	0.0060 (11)	−0.0037 (11)
N2C	0.0398 (16)	0.0254 (13)	0.0254 (13)	0.0066 (11)	0.0122 (11)	0.0015 (10)
N3C	0.0277 (14)	0.0337 (16)	0.0454 (17)	0.0088 (12)	0.0141 (13)	0.0092 (13)
C1C	0.0246 (14)	0.0188 (13)	0.0229 (13)	0.0002 (11)	0.0048 (10)	0.0017 (10)
C2C	0.0250 (14)	0.0163 (13)	0.0285 (14)	0.0005 (10)	0.0086 (11)	0.0031 (11)
C3C	0.0289 (15)	0.0181 (13)	0.0212 (13)	0.0018 (11)	0.0102 (11)	0.0009 (10)
C4C	0.0260 (15)	0.0207 (14)	0.0263 (14)	−0.0002 (11)	0.0067 (11)	0.0037 (11)
C5C	0.0221 (14)	0.0198 (13)	0.0330 (15)	0.0025 (11)	0.0090 (12)	0.0021 (11)
C6C	0.0295 (15)	0.0172 (13)	0.0293 (15)	0.0001 (11)	0.0124 (12)	0.0002 (11)
O1D	0.0479 (16)	0.0509 (17)	0.0263 (12)	−0.0046 (13)	0.0127 (11)	−0.0048 (11)
O2D	0.0306 (14)	0.072 (2)	0.0364 (14)	−0.0052 (14)	−0.0005 (11)	0.0061 (14)
O3D	0.0220 (11)	0.0246 (12)	0.0502 (15)	0.0019 (9)	0.0081 (10)	−0.0052 (10)
O4D	0.069 (3)	0.141 (4)	0.0406 (18)	0.046 (3)	−0.0046 (16)	−0.019 (2)
O5D	0.057 (3)	0.097 (5)	0.0320 (18)	0.043 (3)	0.013 (2)	0.002 (3)
O5DA	0.057 (3)	0.097 (5)	0.0320 (18)	0.043 (3)	0.013 (2)	0.002 (3)
O6D	0.0276 (13)	0.0530 (18)	0.0538 (17)	0.0088 (12)	0.0001 (12)	0.0068 (14)
O7D	0.0404 (16)	0.062 (2)	0.0600 (19)	0.0220 (15)	0.0159 (14)	−0.0066 (16)
N1D	0.0344 (15)	0.0273 (14)	0.0280 (13)	0.0019 (11)	0.0051 (11)	0.0007 (11)
N2D	0.0415 (17)	0.0435 (18)	0.0284 (14)	0.0151 (14)	0.0075 (12)	−0.0011 (13)
N3D	0.0243 (14)	0.0287 (15)	0.0497 (18)	0.0059 (11)	0.0093 (13)	0.0053 (13)
C1D	0.0239 (14)	0.0217 (14)	0.0251 (14)	−0.0012 (11)	0.0044 (11)	0.0002 (11)
C2D	0.0228 (14)	0.0148 (12)	0.0282 (14)	0.0004 (10)	0.0077 (11)	0.0011 (10)
C3D	0.0284 (15)	0.0195 (13)	0.0246 (13)	0.0012 (11)	0.0089 (11)	0.0020 (11)
C4D	0.0273 (15)	0.0208 (14)	0.0298 (15)	−0.0007 (11)	0.0059 (12)	0.0047 (11)
C5D	0.0215 (14)	0.0207 (14)	0.0375 (17)	0.0033 (11)	0.0091 (12)	0.0028 (12)
C6D	0.0279 (15)	0.0176 (13)	0.0325 (16)	0.0014 (11)	0.0099 (12)	−0.0002 (11)
O1E	0.067 (2)	0.0312 (14)	0.0550 (18)	0.0026 (14)	0.0057 (15)	0.0125 (13)
O2E	0.080 (2)	0.0510 (19)	0.0371 (16)	−0.0037 (16)	0.0167 (15)	0.0018 (13)
O3E	0.0479 (16)	0.0327 (13)	0.0366 (13)	−0.0064 (12)	0.0054 (11)	−0.0076 (11)
O4E	0.0493 (17)	0.0308 (14)	0.0586 (18)	−0.0068 (12)	0.0237 (14)	−0.0113 (12)
O5E	0.0592 (19)	0.0434 (17)	0.061 (2)	0.0070 (15)	0.0172 (16)	0.0198 (15)
O6E	0.067 (2)	0.081 (3)	0.0330 (15)	0.0158 (19)	−0.0091 (14)	−0.0059 (16)
O7E	0.0522 (19)	0.060 (2)	0.0538 (18)	−0.0085 (16)	0.0025 (14)	−0.0243 (16)
N1E	0.0383 (16)	0.0356 (16)	0.0351 (16)	0.0012 (13)	0.0059 (13)	0.0061 (13)
N2E	0.0348 (16)	0.0313 (16)	0.054 (2)	0.0106 (13)	0.0216 (14)	0.0077 (14)
N3E	0.0327 (16)	0.062 (2)	0.0362 (17)	0.0096 (16)	0.0003 (13)	−0.0184 (16)
C1E	0.0296 (16)	0.0293 (15)	0.0269 (15)	0.0066 (13)	0.0063 (12)	0.0011 (12)
C2E	0.0275 (15)	0.0266 (15)	0.0315 (16)	0.0070 (12)	0.0068 (12)	−0.0048 (12)
C3E	0.0294 (16)	0.0236 (14)	0.0352 (16)	0.0056 (12)	0.0104 (13)	0.0013 (12)
C4E	0.0294 (16)	0.0405 (19)	0.0279 (16)	0.0141 (14)	0.0077 (12)	0.0052 (13)
C5E	0.0256 (15)	0.0394 (19)	0.0282 (15)	0.0090 (13)	0.0013 (12)	−0.0078 (14)
C6E	0.0251 (15)	0.0279 (16)	0.0404 (18)	0.0034 (12)	0.0072 (13)	−0.0054 (13)

### Geometric parameters (Å, º)

**Table:**

Cl1A—C7A	1.732 (4)	O1C—N1C	1.233 (4)
N1A—H1A	1.0002	O2C—N1C	1.228 (4)
N1A—C1A	1.460 (4)	O3C—C2C	1.258 (4)
N1A—C4A	1.454 (4)	O4C—N2C	1.228 (4)
N1A—C5A	1.405 (4)	O5C—N2C	1.226 (4)
N2A—H2A	0.8805	O6C—N3C	1.219 (4)
N2A—C2A	1.486 (4)	O7C—N3C	1.219 (4)
N2A—C3A	1.487 (4)	N1C—C1C	1.455 (4)
C1A—H1AA	0.9900	N2C—C3C	1.455 (4)
C1A—H1AB	0.9900	N3C—C5C	1.456 (4)
C1A—C2A	1.508 (5)	C1C—C2C	1.447 (4)
C2A—H2AA	0.9900	C1C—C6C	1.374 (4)
C2A—H2AB	0.9900	C2C—C3C	1.446 (4)
C3A—H3AA	0.9900	C3C—C4C	1.392 (4)
C3A—H3AB	0.9900	C4C—H4C	0.9500
C3A—C4A	1.507 (5)	C4C—C5C	1.372 (4)
C4A—H4AA	0.9900	C5C—C6C	1.379 (5)
C4A—H4AB	0.9900	C6C—H6C	0.9500
C5A—C6A	1.401 (5)	O1D—N1D	1.217 (4)
C5A—C10A	1.401 (5)	O2D—N1D	1.232 (4)
C6A—H6A	0.9500	O3D—C2D	1.254 (4)
C6A—C7A	1.391 (5)	O4D—N2D	1.201 (5)
C7A—C8A	1.376 (6)	O5D—N2D	1.267 (6)
C8A—H8A	0.9500	O5DA—N2D	1.230 (11)
C8A—C9A	1.385 (7)	O6D—N3D	1.227 (4)
C9A—H9A	0.9500	O7D—N3D	1.225 (4)
C9A—C10A	1.386 (5)	N1D—C1D	1.456 (4)
C10A—H10A	0.9500	N2D—C3D	1.458 (4)
Cl1B—C7B	1.748 (3)	N3D—C5D	1.460 (4)
N1B—H1B	1.0002	C1D—C2D	1.441 (4)
N1B—C1B	1.459 (4)	C1D—C6D	1.378 (4)
N1B—C4B	1.459 (4)	C2D—C3D	1.444 (4)
N1B—C5B	1.398 (4)	C3D—C4D	1.377 (4)
N2B—H2B	0.8797	C4D—H4D	0.9500
N2B—C2B	1.479 (4)	C4D—C5D	1.383 (5)
N2B—C3B	1.492 (4)	C5D—C6D	1.378 (5)
C1B—H1BA	0.9900	C6D—H6D	0.9500
C1B—H1BB	0.9900	O1E—N1E	1.233 (4)
C1B—C2B	1.525 (5)	O2E—N1E	1.200 (4)
C2B—H2BA	0.9900	O3E—H3E	0.8395
C2B—H2BB	0.9900	O3E—C2E	1.319 (4)
C3B—H3BA	0.9900	O4E—N2E	1.238 (5)
C3B—H3BB	0.9900	O5E—N2E	1.218 (4)
C3B—C4B	1.511 (5)	O6E—N3E	1.221 (5)
C4B—H4BA	0.9900	O7E—N3E	1.220 (5)
C4B—H4BB	0.9900	N1E—C1E	1.467 (4)
C5B—C6B	1.410 (4)	N2E—C3E	1.461 (5)
C5B—C10B	1.404 (4)	N3E—C5E	1.472 (5)
C6B—H6B	0.9500	C1E—C2E	1.407 (5)
C6B—C7B	1.382 (4)	C1E—C6E	1.378 (5)
C7B—C8B	1.381 (5)	C2E—C3E	1.407 (5)
C8B—H8B	0.9500	C3E—C4E	1.390 (5)
C8B—C9B	1.392 (5)	C4E—H4E	0.9500
C9B—H9B	0.9500	C4E—C5E	1.380 (6)
C9B—C10B	1.384 (5)	C5E—C6E	1.375 (5)
C10B—H10B	0.9500	C6E—H6E	0.9500
			
C1A—N1A—H1A	101.2	C7B—C8B—H8B	121.6
C4A—N1A—H1A	101.1	C7B—C8B—C9B	116.8 (3)
C4A—N1A—C1A	113.2 (3)	C9B—C8B—H8B	121.6
C5A—N1A—H1A	101.1	C8B—C9B—H9B	119.3
C5A—N1A—C1A	118.2 (3)	C10B—C9B—C8B	121.5 (3)
C5A—N1A—C4A	117.7 (3)	C10B—C9B—H9B	119.3
C2A—N2A—H2A	124.9	C5B—C10B—H10B	119.3
C2A—N2A—C3A	110.0 (3)	C9B—C10B—C5B	121.5 (3)
C3A—N2A—H2A	125.1	C9B—C10B—H10B	119.3
N1A—C1A—H1AA	109.3	O1C—N1C—C1C	117.5 (3)
N1A—C1A—H1AB	109.3	O2C—N1C—O1C	123.3 (3)
N1A—C1A—C2A	111.8 (3)	O2C—N1C—C1C	119.2 (3)
H1AA—C1A—H1AB	107.9	O4C—N2C—C3C	119.8 (3)
C2A—C1A—H1AA	109.3	O5C—N2C—O4C	122.2 (3)
C2A—C1A—H1AB	109.3	O5C—N2C—C3C	118.0 (3)
N2A—C2A—C1A	110.2 (3)	O6C—N3C—O7C	123.5 (3)
N2A—C2A—H2AA	109.6	O6C—N3C—C5C	118.7 (3)
N2A—C2A—H2AB	109.6	O7C—N3C—C5C	117.7 (3)
C1A—C2A—H2AA	109.6	C2C—C1C—N1C	119.1 (3)
C1A—C2A—H2AB	109.6	C6C—C1C—N1C	116.0 (3)
H2AA—C2A—H2AB	108.1	C6C—C1C—C2C	124.9 (3)
N2A—C3A—H3AA	109.3	O3C—C2C—C1C	122.7 (3)
N2A—C3A—H3AB	109.3	O3C—C2C—C3C	125.5 (3)
N2A—C3A—C4A	111.8 (3)	C3C—C2C—C1C	111.7 (3)
H3AA—C3A—H3AB	107.9	C2C—C3C—N2C	120.9 (3)
C4A—C3A—H3AA	109.3	C4C—C3C—N2C	115.1 (3)
C4A—C3A—H3AB	109.3	C4C—C3C—C2C	123.9 (3)
N1A—C4A—C3A	112.4 (3)	C3C—C4C—H4C	120.6
N1A—C4A—H4AA	109.1	C5C—C4C—C3C	118.9 (3)
N1A—C4A—H4AB	109.1	C5C—C4C—H4C	120.6
C3A—C4A—H4AA	109.1	C4C—C5C—N3C	118.8 (3)
C3A—C4A—H4AB	109.1	C4C—C5C—C6C	122.0 (3)
H4AA—C4A—H4AB	107.8	C6C—C5C—N3C	119.1 (3)
C6A—C5A—N1A	120.8 (3)	C1C—C6C—C5C	118.6 (3)
C6A—C5A—C10A	117.9 (3)	C1C—C6C—H6C	120.7
C10A—C5A—N1A	121.3 (3)	C5C—C6C—H6C	120.7
C5A—C6A—H6A	120.2	O1D—N1D—O2D	123.3 (3)
C7A—C6A—C5A	119.5 (3)	O1D—N1D—C1D	118.0 (3)
C7A—C6A—H6A	120.2	O2D—N1D—C1D	118.6 (3)
C6A—C7A—Cl1A	118.4 (3)	O4D—N2D—O5D	116.2 (4)
C8A—C7A—Cl1A	118.9 (3)	O4D—N2D—O5DA	116.5 (6)
C8A—C7A—C6A	122.7 (4)	O4D—N2D—C3D	119.6 (3)
C7A—C8A—H8A	121.2	O5D—N2D—C3D	118.8 (3)
C7A—C8A—C9A	117.6 (3)	O5DA—N2D—C3D	116.5 (5)
C9A—C8A—H8A	121.2	O6D—N3D—C5D	118.2 (3)
C8A—C9A—H9A	119.3	O7D—N3D—O6D	123.7 (3)
C8A—C9A—C10A	121.4 (4)	O7D—N3D—C5D	118.1 (3)
C10A—C9A—H9A	119.3	C2D—C1D—N1D	118.4 (3)
C5A—C10A—H10A	119.6	C6D—C1D—N1D	116.8 (3)
C9A—C10A—C5A	120.9 (4)	C6D—C1D—C2D	124.6 (3)
C9A—C10A—H10A	119.6	O3D—C2D—C1D	123.4 (3)
C1B—N1B—H1B	101.6	O3D—C2D—C3D	124.7 (3)
C4B—N1B—H1B	101.7	C1D—C2D—C3D	111.9 (3)
C4B—N1B—C1B	112.8 (3)	C2D—C3D—N2D	119.4 (3)
C5B—N1B—H1B	101.6	C4D—C3D—N2D	116.2 (3)
C5B—N1B—C1B	117.6 (2)	C4D—C3D—C2D	124.4 (3)
C5B—N1B—C4B	117.8 (2)	C3D—C4D—H4D	120.6
C2B—N2B—H2B	124.7	C3D—C4D—C5D	118.8 (3)
C2B—N2B—C3B	110.6 (3)	C5D—C4D—H4D	120.6
C3B—N2B—H2B	124.7	C4D—C5D—N3D	119.5 (3)
N1B—C1B—H1BA	109.3	C6D—C5D—N3D	118.7 (3)
N1B—C1B—H1BB	109.3	C6D—C5D—C4D	121.7 (3)
N1B—C1B—C2B	111.7 (3)	C1D—C6D—C5D	118.7 (3)
H1BA—C1B—H1BB	107.9	C1D—C6D—H6D	120.7
C2B—C1B—H1BA	109.3	C5D—C6D—H6D	120.7
C2B—C1B—H1BB	109.3	C2E—O3E—H3E	109.5
N2B—C2B—C1B	111.2 (3)	O1E—N1E—C1E	116.5 (3)
N2B—C2B—H2BA	109.4	O2E—N1E—O1E	123.9 (3)
N2B—C2B—H2BB	109.4	O2E—N1E—C1E	119.6 (3)
C1B—C2B—H2BA	109.4	O4E—N2E—C3E	118.6 (3)
C1B—C2B—H2BB	109.4	O5E—N2E—O4E	123.1 (4)
H2BA—C2B—H2BB	108.0	O5E—N2E—C3E	118.2 (3)
N2B—C3B—H3BA	109.7	O6E—N3E—C5E	116.7 (4)
N2B—C3B—H3BB	109.7	O7E—N3E—O6E	125.4 (4)
N2B—C3B—C4B	110.0 (3)	O7E—N3E—C5E	117.9 (4)
H3BA—C3B—H3BB	108.2	C2E—C1E—N1E	120.9 (3)
C4B—C3B—H3BA	109.7	C6E—C1E—N1E	116.1 (3)
C4B—C3B—H3BB	109.7	C6E—C1E—C2E	123.0 (3)
N1B—C4B—C3B	111.3 (3)	O3E—C2E—C1E	119.0 (3)
N1B—C4B—H4BA	109.4	O3E—C2E—C3E	125.2 (3)
N1B—C4B—H4BB	109.4	C1E—C2E—C3E	115.7 (3)
C3B—C4B—H4BA	109.4	C2E—C3E—N2E	119.7 (3)
C3B—C4B—H4BB	109.4	C4E—C3E—N2E	117.6 (3)
H4BA—C4B—H4BB	108.0	C4E—C3E—C2E	122.7 (3)
N1B—C5B—C6B	121.3 (3)	C3E—C4E—H4E	121.1
N1B—C5B—C10B	121.7 (3)	C5E—C4E—C3E	117.8 (3)
C10B—C5B—C6B	117.0 (3)	C5E—C4E—H4E	121.1
C5B—C6B—H6B	120.0	C4E—C5E—N3E	119.3 (3)
C7B—C6B—C5B	119.9 (3)	C6E—C5E—N3E	118.1 (4)
C7B—C6B—H6B	120.0	C6E—C5E—C4E	122.7 (3)
C6B—C7B—Cl1B	118.3 (3)	C1E—C6E—H6E	120.9
C8B—C7B—Cl1B	118.4 (3)	C5E—C6E—C1E	118.1 (3)
C8B—C7B—C6B	123.3 (3)	C5E—C6E—H6E	120.9
			
Cl1A—C7A—C8A—C9A	−179.3 (3)	C2C—C1C—C6C—C5C	−1.1 (5)
N1A—C1A—C2A—N2A	−56.5 (4)	C2C—C3C—C4C—C5C	−0.6 (5)
N1A—C5A—C6A—C7A	−174.9 (3)	C3C—C4C—C5C—N3C	−179.2 (3)
N1A—C5A—C10A—C9A	175.7 (3)	C3C—C4C—C5C—C6C	−0.9 (5)
N2A—C3A—C4A—N1A	52.1 (5)	C4C—C5C—C6C—C1C	1.7 (5)
C1A—N1A—C4A—C3A	−50.7 (4)	C6C—C1C—C2C—O3C	−178.3 (3)
C1A—N1A—C5A—C6A	−160.8 (3)	C6C—C1C—C2C—C3C	−0.2 (4)
C1A—N1A—C5A—C10A	21.6 (4)	O1D—N1D—C1D—C2D	142.0 (3)
C2A—N2A—C3A—C4A	−55.7 (4)	O1D—N1D—C1D—C6D	−33.1 (4)
C3A—N2A—C2A—C1A	57.6 (4)	O2D—N1D—C1D—C2D	−39.0 (4)
C4A—N1A—C1A—C2A	53.2 (4)	O2D—N1D—C1D—C6D	145.9 (3)
C4A—N1A—C5A—C6A	−19.0 (4)	O3D—C2D—C3D—N2D	−1.9 (5)
C4A—N1A—C5A—C10A	163.4 (3)	O3D—C2D—C3D—C4D	−179.9 (3)
C5A—N1A—C1A—C2A	−163.4 (3)	O4D—N2D—C3D—C2D	163.1 (4)
C5A—N1A—C4A—C3A	165.7 (3)	O4D—N2D—C3D—C4D	−18.7 (6)
C5A—C6A—C7A—Cl1A	177.6 (3)	O5D—N2D—C3D—C2D	10.1 (6)
C5A—C6A—C7A—C8A	−2.2 (5)	O5D—N2D—C3D—C4D	−171.8 (5)
C6A—C5A—C10A—C9A	−2.0 (5)	O5DA—N2D—C3D—C2D	−47.9 (8)
C6A—C7A—C8A—C9A	0.6 (6)	O5DA—N2D—C3D—C4D	130.2 (8)
C7A—C8A—C9A—C10A	0.3 (6)	O6D—N3D—C5D—C4D	−4.1 (5)
C8A—C9A—C10A—C5A	0.4 (6)	O6D—N3D—C5D—C6D	172.6 (3)
C10A—C5A—C6A—C7A	2.8 (5)	O7D—N3D—C5D—C4D	177.9 (3)
Cl1B—C7B—C8B—C9B	179.7 (3)	O7D—N3D—C5D—C6D	−5.4 (5)
N1B—C1B—C2B—N2B	−53.1 (4)	N1D—C1D—C2D—O3D	4.1 (4)
N1B—C5B—C6B—C7B	177.5 (3)	N1D—C1D—C2D—C3D	−173.8 (3)
N1B—C5B—C10B—C9B	−176.9 (3)	N1D—C1D—C6D—C5D	175.7 (3)
N2B—C3B—C4B—N1B	57.0 (4)	N2D—C3D—C4D—C5D	−176.9 (3)
C1B—N1B—C4B—C3B	−55.3 (4)	N3D—C5D—C6D—C1D	−178.5 (3)
C1B—N1B—C5B—C6B	16.1 (4)	C1D—C2D—C3D—N2D	176.0 (3)
C1B—N1B—C5B—C10B	−166.7 (3)	C1D—C2D—C3D—C4D	−2.0 (4)
C2B—N2B—C3B—C4B	−57.5 (4)	C2D—C1D—C6D—C5D	1.0 (5)
C3B—N2B—C2B—C1B	55.7 (4)	C2D—C3D—C4D—C5D	1.1 (5)
C4B—N1B—C1B—C2B	53.0 (4)	C3D—C4D—C5D—N3D	177.5 (3)
C4B—N1B—C5B—C6B	156.3 (3)	C3D—C4D—C5D—C6D	1.0 (5)
C4B—N1B—C5B—C10B	−26.5 (4)	C4D—C5D—C6D—C1D	−2.0 (5)
C5B—N1B—C1B—C2B	−164.9 (3)	C6D—C1D—C2D—O3D	178.8 (3)
C5B—N1B—C4B—C3B	162.6 (3)	C6D—C1D—C2D—C3D	0.9 (4)
C5B—C6B—C7B—Cl1B	179.6 (2)	O1E—N1E—C1E—C2E	−150.7 (3)
C5B—C6B—C7B—C8B	0.0 (5)	O1E—N1E—C1E—C6E	30.0 (5)
C6B—C5B—C10B—C9B	0.4 (5)	O2E—N1E—C1E—C2E	31.7 (5)
C6B—C7B—C8B—C9B	−0.7 (5)	O2E—N1E—C1E—C6E	−147.7 (4)
C7B—C8B—C9B—C10B	1.3 (5)	O3E—C2E—C3E—N2E	1.8 (5)
C8B—C9B—C10B—C5B	−1.1 (5)	O3E—C2E—C3E—C4E	179.6 (3)
C10B—C5B—C6B—C7B	0.1 (4)	O4E—N2E—C3E—C2E	−1.2 (5)
O1C—N1C—C1C—C2C	−144.5 (3)	O4E—N2E—C3E—C4E	−179.1 (3)
O1C—N1C—C1C—C6C	32.3 (4)	O5E—N2E—C3E—C2E	177.7 (3)
O2C—N1C—C1C—C2C	36.0 (4)	O5E—N2E—C3E—C4E	−0.1 (5)
O2C—N1C—C1C—C6C	−147.3 (3)	O6E—N3E—C5E—C4E	−6.5 (5)
O3C—C2C—C3C—N2C	0.6 (5)	O6E—N3E—C5E—C6E	172.4 (3)
O3C—C2C—C3C—C4C	179.1 (3)	O7E—N3E—C5E—C4E	173.8 (3)
O4C—N2C—C3C—C2C	−4.5 (5)	O7E—N3E—C5E—C6E	−7.2 (5)
O4C—N2C—C3C—C4C	177.0 (3)	N1E—C1E—C2E—O3E	2.9 (5)
O5C—N2C—C3C—C2C	175.3 (3)	N1E—C1E—C2E—C3E	−179.2 (3)
O5C—N2C—C3C—C4C	−3.3 (4)	N1E—C1E—C6E—C5E	177.1 (3)
O6C—N3C—C5C—C4C	15.4 (5)	N2E—C3E—C4E—C5E	176.3 (3)
O6C—N3C—C5C—C6C	−162.9 (3)	N3E—C5E—C6E—C1E	−176.3 (3)
O7C—N3C—C5C—C4C	−166.0 (4)	C1E—C2E—C3E—N2E	−176.0 (3)
O7C—N3C—C5C—C6C	15.7 (5)	C1E—C2E—C3E—C4E	1.8 (5)
N1C—C1C—C2C—O3C	−1.9 (4)	C2E—C1E—C6E—C5E	−2.3 (5)
N1C—C1C—C2C—C3C	176.2 (3)	C2E—C3E—C4E—C5E	−1.5 (5)
N1C—C1C—C6C—C5C	−177.6 (3)	C3E—C4E—C5E—N3E	178.1 (3)
N2C—C3C—C4C—C5C	177.9 (3)	C3E—C4E—C5E—C6E	−0.8 (5)
N3C—C5C—C6C—C1C	180.0 (3)	C4E—C5E—C6E—C1E	2.6 (5)
C1C—C2C—C3C—N2C	−177.4 (3)	C6E—C1E—C2E—O3E	−177.8 (3)
C1C—C2C—C3C—C4C	1.1 (4)	C6E—C1E—C2E—C3E	0.2 (5)

### Hydrogen-bond geometry (Å, º)

**Table:**

*D*—H···*A*	*D*—H	H···*A*	*D*···*A*	*D*—H···*A*
N1*A*—H1*A*···Cl1*B*^i^	1.00	2.78	3.665 (3)	147
N2*A*—H2*A*···O3*C*	0.88	2.29	2.723 (4)	110
N2*A*—H2*A*···O3*D*	0.88	2.34	2.787 (4)	112
C3*A*—H3*AB*···O7*D*^ii^	0.99	2.63	3.451 (5)	140
C4*A*—H4*AA*···Cl1*B*^iii^	0.99	2.88	3.690 (4)	139
C4*A*—H4*AB*···O2*D*	0.99	2.51	3.253 (5)	132
C8*A*—H8*A*···O7*C*^iv^	0.95	2.64	3.572 (5)	168
C10*A*—H10*A*···O6*E*^v^	0.95	2.53	3.400 (5)	152
N1*B*—H1*B*···Cl1*A*^vi^	1.00	2.80	3.653 (3)	143
N2*B*—H2*B*···O3*C*	0.88	2.38	2.848 (4)	114
N2*B*—H2*B*···O3*D*	0.88	2.34	2.771 (4)	111
C1*B*—H1*BA*···O2*C*	0.99	2.66	3.375 (5)	129
C1*B*—H1*BB*···O2*D*^vii^	0.99	2.52	3.317 (4)	137
C2*B*—H2*BA*···O7*C*^viii^	0.99	2.56	3.419 (5)	145
C4*B*—H4*BA*···O1*C*^iii^	0.99	2.63	3.232 (4)	120
C6*C*—H6*C*···O5*C*^vii^	0.95	2.48	3.310 (4)	146
O3*E*—H3*E*···O4*C*	0.84	2.51	3.037 (4)	122
O3*E*—H3*E*···O5*C*	0.84	2.43	2.962 (4)	122
O3*E*—H3*E*···O4*E*	0.84	1.86	2.565 (4)	140
O3*E*—H3*E*···N2*E*	0.84	2.47	2.899 (4)	113
C4*E*—H4*E*···O4*D*^ix^	0.95	2.57	3.412 (6)	148

Symmetry codes: (i) *x*, *y*−1, *z*; (ii) *x*−1, *y*, *z*; (iii) *x*, −*y*+2, *z*−1/2; (iv) *x*+1, *y*−1, *z*; (v) *x*+1, *y*, *z*+1; (vi) *x*, *y*+1, *z*; (vii) *x*, −*y*+2, *z*+1/2; (viii) *x*+1, *y*, *z*; (ix) *x*−1, *y*, *z*−1.
