# Supplementary figures and images for: Crystal structure of 1-(3-chloro­phen­yl)piperazin-1-ium picrate–picric acid (2/1)
Source: Acta Crystallogr Sect E Struct Rep Online. 2014 Oct 31;70(Pt 11):o1210–1. doi: 10.1107/S1600536814023654 (PMC4257327; doi:10.1107/S1600536814023654)

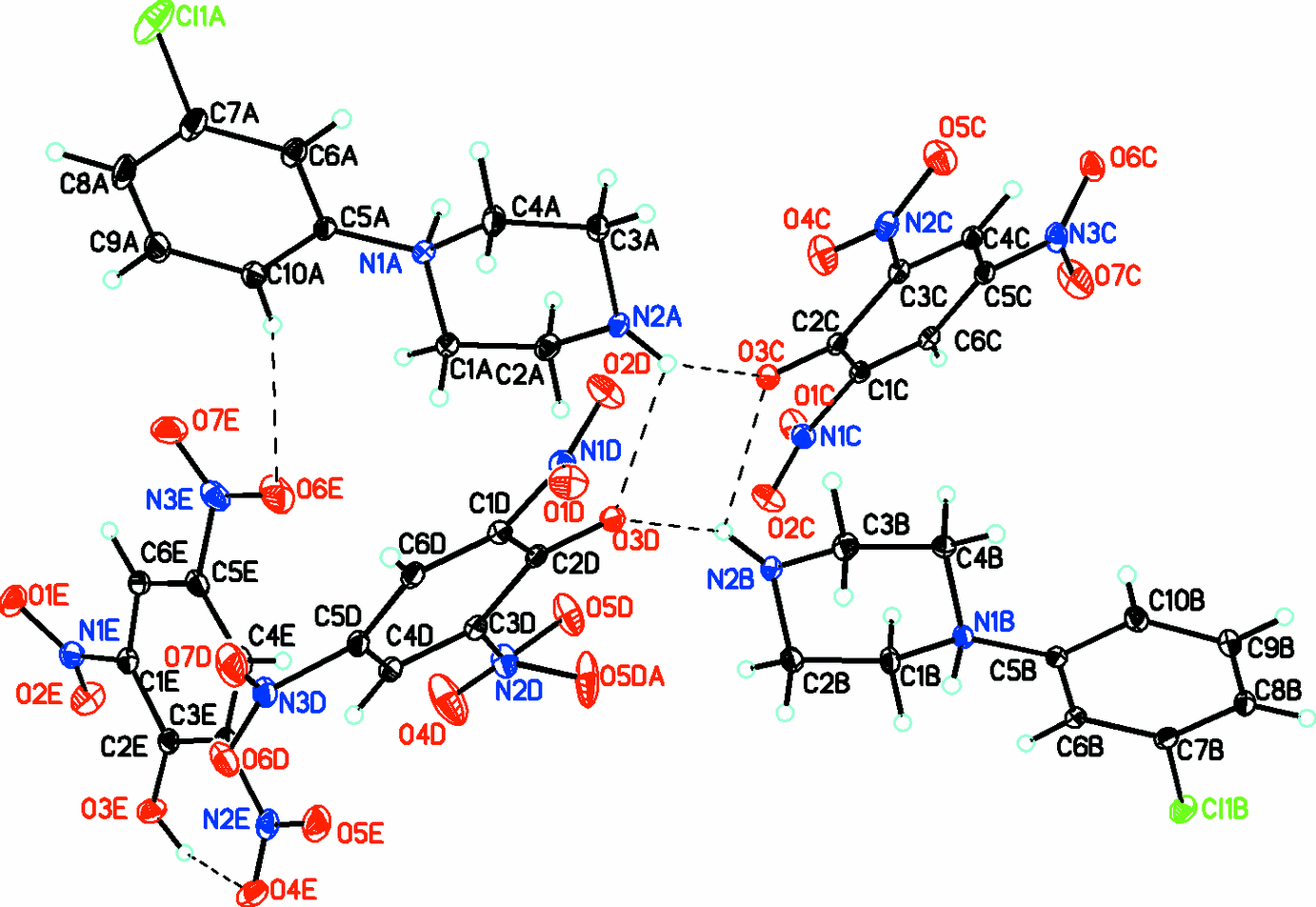

Supplement: Supplementary file 4 [file e-70-o1210-fig1.tif]

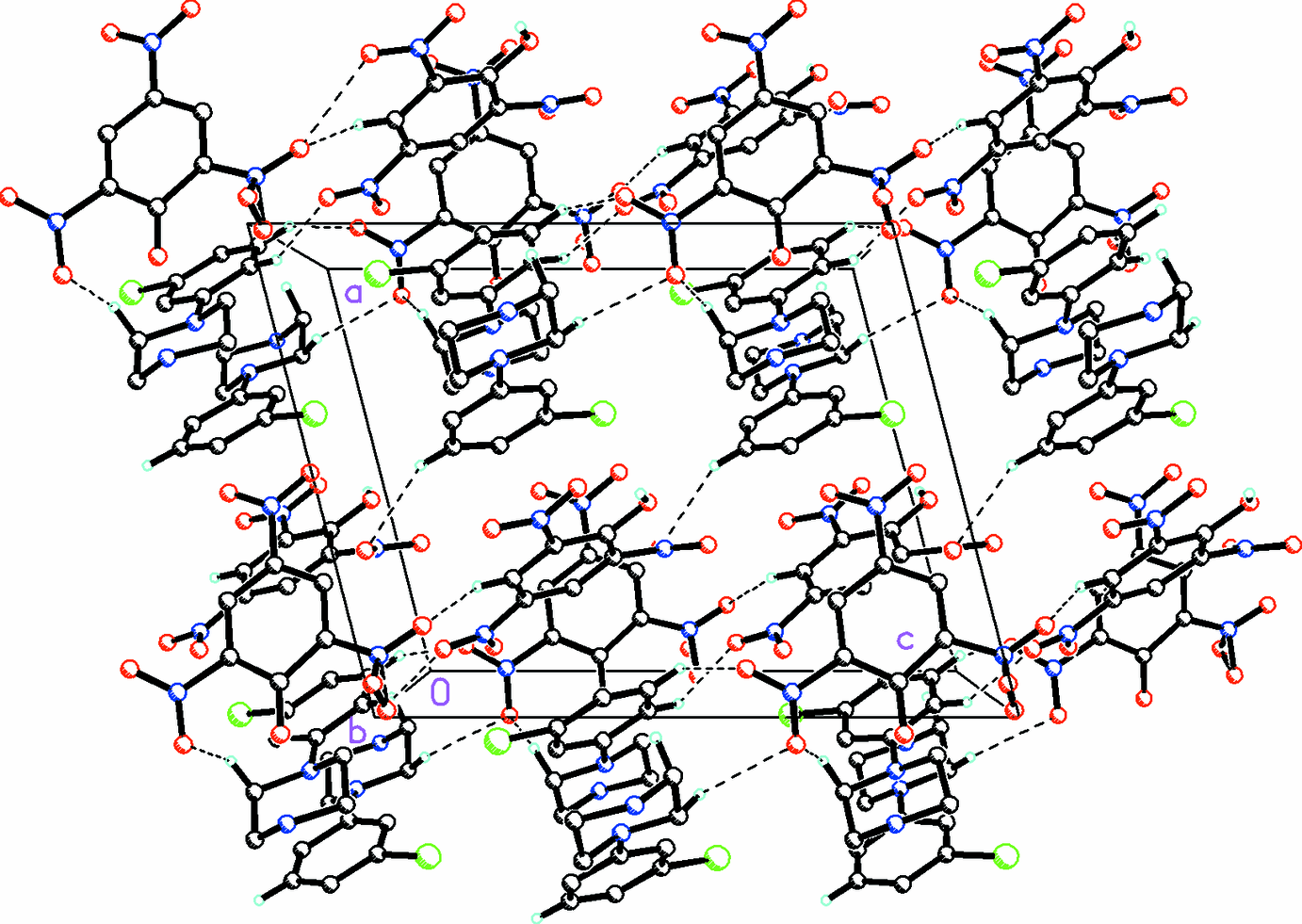

Supplement: Supplementary file 5 [file e-70-o1210-fig2.tif]
